# Relaxing Gaze Aversion of Adolescents With Autism Spectrum Disorder in Consecutive Conversations With Human and Android Robot—A Preliminary Study

**DOI:** 10.3389/fpsyt.2019.00370

**Published:** 2019-06-14

**Authors:** Yuichiro Yoshikawa, Hirokazu Kumazaki, Yoshio Matsumoto, Masutomo Miyao, Mitsuru Kikuchi, Hiroshi Ishiguro

**Affiliations:** ^1^Department of Systems Innovation, Graduate School of Engineering Science, Osaka University, Osaka, Japan; ^2^ERATO ISHIGURO Human-Robot Symbiotic Interaction Project, JST, Osaka, Japan; ^3^Department of Clinical Research on Social Recognition and Memory, Research Center for Child Mental Development, Kanazawa University, Ishikawa, Japan; ^4^Department of Preventive Intervention for Psychiatric Disorders, National Institute of Mental Health, National Center of Neurology and Psychiatry, Tokyo, Japan; ^5^Service Robotics Research Group, Intelligent Systems Institute, National Institute of Advanced Industrial Science and Technology, Ibaraki, Japan; ^6^Donguri Psycho Developmental Clinic, Tokyo, Japan

**Keywords:** autism spectrum disorder, eye contact, treatment and education, android robot, eye-gaze tracking

## Abstract

Establishing a treatment method for individuals with autism spectrum disorder (ASD) not only to increase their frequency or duration of eye contact but also to maintain it after ceasing the intervention, and furthermore generalize it across communication partners, is a formidable challenge. Android robots, which are a type of humanoid robot with appearances quite similar to that of humans, are expected to adapt to the role of training partners of face-to-face communication for individuals with ASD and to create easier experiences transferrable to humans. To evaluate this possibility, four male adolescents with ASD and six without ASD were asked to participate a pilot experiment in which there were consecutive sessions of semistructured conversation where they alternately faced either a human female or a female-type android robot interlocutor five times in total. Although it is limited by the small sample size, the preliminary results of analysis of their fixation pattern during the conversations indicated positive signs; the subjects tended to look more at the face of the android robot than that of the human interlocutor regardless of whether they had ASD. However, the individuals with ASD looked more at the area around the eyes of the android robot than at the human, and also looked less at that of the human than the individuals without ASD did. An increasing tendency of looking at the area around the human eyes, which could be a positive sign of the transferability of the experiences with an android robot to a human interlocutor, was only weakly observed as the sessions progressed.

## Introduction

Autism spectrum disorder (ASD) is a neurodevelopmental disorder that includes persistent deficits in social communication across multiple contexts. As presented in a recent statistical report (1), the necessity of treatment and education for children and adolescents with autism spectrum disorder (ASD) has been widely recognized. It has been reported that persons with ASD pay less attention to the area of the eyes in the static pictures of a human face than persons with typical development (2). In particular, adolescents and children with ASD have been shown to spend significantly less time fixating on the eyes of persons on static pictures (3) and dynamic audiovisual stimuli (4), respectively. Additionally, children with ASD are known to look down more often and explore the lateral field of view in semistructured live interactions, which probably reflects their wish to view static stimuli that will not perturb them (5). Accordingly, absent, reduced, or atypical use of eye contact is considered to be one of the diagnostic features of ASD, manifesting the deficits in nonverbal communicative behaviors used for social interaction (6). Nevertheless, it is one of the most important cues for communication (7). Although increasing eye contact is widely acknowledged as an important and promising treatment for children with ASD (8, 9), there is no reliable and established procedures not only to increase it but also to maintain it after ceasing the intervention, and furthermore generalize it across communication partners.

Recent advanced robot technology may enable us to think of clinical applications using robots for ASD. Previous studies suggested that children or adolescents with ASD could show social or positive attitudes toward robots, and based on that, their social development can be hopefully guided (10–14). It has been attempted to evaluate the effects of a prolonged intervention using a small humanoid robot and a mobile robot on various aspects of behavior related to social communication such as social attention (15), verbal communication (16), imitation and synchrony (17), and sensory behavior and affective states (18). However, it has not been still clear whether or what kinds of the intervention using robots are more effective to support acquiring and generalizing them compared to the interventions by humans. A humanoid robot is a type of robot with a body structure similar to that of a human. Its artificial human likeness is expected to provide individuals with ASD with easy and less stressful opportunities to experience social interaction because it is at present still difficult to implement its nonverbal behavior such as eye contact in a manner that matches that of humans. Furthermore, such opportunities are expected to enable individuals with ASD to become accustomed to communication using eyes and enable them to establish more successful social communication with others. It has been reported that a small humanoid robot could establish eye contact with children with ASD more frequently than a human therapist during initial sessions of training for recognizing facial expressions (19). However, it was also reported that the frequency of eye contact did not significantly change between sessions of the joint interactive play task of Autism Diagnostic Observation Schedule (ADOS) measured before and after training sessions. Although small humanoid robots have succeeded in teaching robotic social cues such as head-gaze and hand-pointing (11), they have not been generalized for interactions with humans.

One possible reason for this might be insufficiency of human likeness of the robot used in the previous work. It is necessary to find a sufficiently acceptable and influential robot design. In this study, we therefore started basic investigations of how adolescents with ASD respond to a special type of robot called an android robot. An android robot is a type of humanoid robot that has appearance resembling a real person and has recently been focused on as an influential information media for humans (20). Because their appearance is quite similar to that of humans, it is expected that they could perform the role of training partners or instructors to teach social skills and protocols, and to create easier experiences that are transferrable to humans.

As a first step in designing transferrable experiences to human, it is necessary to evaluate whether it is easier for individuals with ASD to look at the eyes of an android robot than those of a person during face-to-face conversation. Therefore, we made a robot system using a female-type android that can face a subject and conduct a semistructured conversation. In this study, to focus on the relatively instant effects of the interaction with the android robot as the first step, the conversation sessions were conducted in 1 day rather than considering multiday or multiweek intervention as some previous studies have concerned. Although it should be treated as a pilot experiment due to the small sample size, subjects with and without ASD participated in consecutive sessions where they alternately talked to the human and android interlocutors five times in total. Eye-tracker devices were used to detect the subjects’ fixation points during the conversations to analyze the tendency of the looking pattern of individuals with and without ASD when they faced the human or the android robot. Further, evaluations were conducted of whether the tendency of the looking pattern for the human interlocutor changed along with the extension of the session to argue the potential of human–robot conversation as a method for treatment and education of communication with humans using eyes.

## Materials and Methods

### Participants

The current study was approved by the ethics committee of University of Fukui. Written informed consent was obtained from all participants included in the study and their guardians. On the day of the experiment, a teacher of a school for students with special needs showed students the android robot, explained the conversation experiment to be undertaken with it, and requested volunteers to participate in the experiment. Then, the experienced medical doctors (the second and fourth authors) confirmed that none of the participants had any severe language disability.

Four male adolescents with ASD participated in the experiment. The inclusion criteria were that participants should be between 15 and 18 years and have a previous diagnosis of ASD. They had previously received a clinical diagnosis of ASD based on the *Diagnostic and Statistical Manual of Mental Disorders, Fifth Edition* (DSM-5) (6) and were further diagnosed through the consensus of a clinical team comprising experienced professionals (child and adolescent psychiatrist, clinical psychologist, and pediatric neurologist). The team assessments were made following a detailed clinical examination on the first visit, follow-up observations, and through evaluation of the answers provided in response to a questionnaire related to the development and symptoms of participants, as completed by guardians. Clinical psychologists collected information from guardians concerning developmental milestones (including joint attention, social interaction, pretend play, and repetitive behaviors, with onset prior to 3 years of age) and episodes (e.g., how the individual with ASD behaved in kindergarten and school). Additional professionals, such as teachers, provided further background based on their detailed observations of interactions with people (particularly nonfamily members), repetitive behaviors, obsessive/compulsive traits, and stereotyped behaviors. The second and fourth authors confirmed existing diagnoses by using both diagnostic instruments and screening questionnaires, including the Pervasive Developmental Disorder–Autism Society Japan Rating Scale (PARS), a diagnostic interview scale for ASD developed in Japan (21). Sub- and total scores of this scale correlate with the domain and total scores of the Autism Diagnostic Interview-Revised (ADI-R) (22, 23). To exclude other psychiatric diagnoses, the Mini-International Neuropsychiatric Interview for Children and Adolescents (MINI Kids) (24) was administered.

Six male adolescents without ASD participated in the experiment and were assessed by the same clinical team and in the same way as subjects with ASD. To screen control participants for autistic traits, the Childhood Autism Rating Scale–Tokyo Version (CARS-TV) was used for both groups of participants. The CARS-TV is the Japanese version of the CARS (25)—one of the most widely used scales to evaluate the degree and profiles of autism in children—and has been determined to have satisfactory reliability and validity (26, 27). The scores of CARS for these six participants were lower than the cutoff threshold for diagnosis as ASD, whereas those for the four participants in the ASD group were higher than the cutoff threshold. To exclude other psychiatric diagnoses, MINI Kids was administered. Although the participants in the control group had no ASD or other neuropsychiatric symptoms, they each had a similar history of difficulty adapting to school as those in the ASD group.

### Apparatus

**Figure 1:**
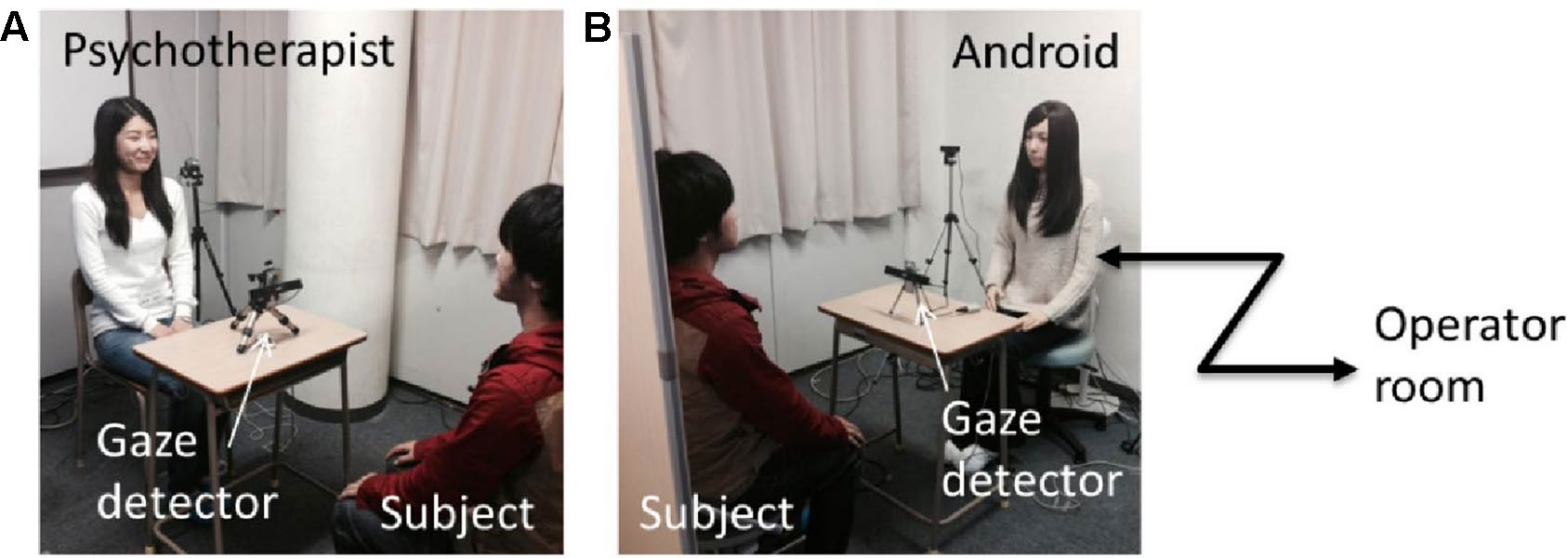
Experimental setup: Human **(A)** and android **(B)** rooms. In both rooms, a gaze detection device was placed on a table between the subject and the interlocutor (human or android robot). The computer interface for the operator to control the android was placed behind the android room. Note that the person labeled as a subject is not a participant included in this study. Note that the written informed consents to publish this figure are obtained from persons who appear in the figures.

Two experimental booths were situated adjacent to each other (see **Figure 1**): the human room for communication with a female person and the android room for communication with a female-type android robot called Actroid-F (Kokoro Co., Ltd). The individual in this manuscript has given written informed consent to publish these case details. The android robot achieved the same appearance of a real individual by making a plaster cast of the person and behaving like humans by using pneumatic actuators to silently and rapidly move its skin. It has 11 degrees of freedom: neck (3), eyeballs (2), eyelids (1), cheek (1), lip (1), eyebrow (2), and bow (1). The utterances were produced by playing voice sounds from a speaker located close to it (note that the voice was prerecorded from the person who also played the role of interlocutor in the human room). Further, it produced spontaneous eye-blinking behavior and mouth open-close movement synchronized with its utterances (note that any facial expression such as smiling and gaze movements as if in a thinking mood were intentionally not implemented to reduce the humanly features of the android) (see **Supplementary Video** for how the android behaved).

The utterances of the human interlocutor and the android interlocutor were scripted in advance. In each script, they asked questions of the participants, waited for a while, and then commented on the participants’ answers. The questions and comments in the scripts were carefully chosen such that they could maintain consistency in the conversation after receiving various possible adolescents’ answers. In other words, participants’ experiences were designed to be interactive as well as equivalent among participants. Different scripts were prepared for different sessions to avoid boring the subjects. The first human session and the first android session included questions regarding the subject as well as questions regarding the current interlocutor. The second and third human sessions and the second android session included questions regarding the opposite interlocutor. The android was operated based on the Wizard of Oz technique. Instead of using error-prone automatic functions to judge the end of the utterance of the subject, the timing to produce the next utterance was judged by a tele-operator monitoring the conversation between the subject and the android robot. The system to control the android and the Graphical User Interface (GUI) for the operator was installed in the space behind the rooms and concealed from participants by using wall partitions.

**Figure 2:**
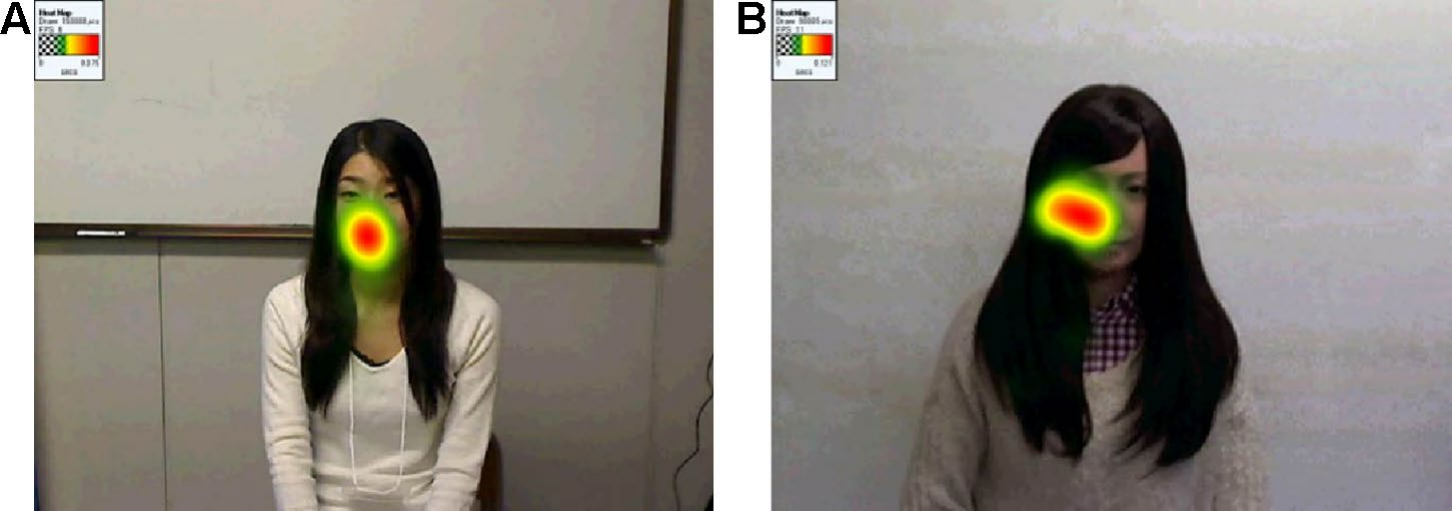
Example visualization of fixation points during conversation with human **(A)** and android **(B)** interlocutors. Color map indicates where the subject likely looks at. Note that the written informed consent to publish this figure is obtained from the person who appears in the figure.

In each booth, an eye-tracker device (Tobii, X2-60) was set to detect the fixation points of the participants during the conversations (see **Figure 2**). Before starting the trials, each device was calibrated to output the participant’s fixation points on a virtual screen located in the position of the human’s or the android’s face, which corresponded to the image plane captured by a video camera behind the participant. The data were processed by analyzing when the detected fixation points stayed on the human’s or android’s face region in the captured images: when they looked at the interlocutor’s face. The area of interest (AOI), that is, the facial region of the interlocutors, was identified around their face using a simple image processing program. The size of the ellipsoid was selected to well fit human and android faces in the recorded video. Then, for every 20 frames of the 30-Hz video stream, we manually clicked the points of facial region to decide on the ellipsoid position. The facial regions appearing in between these frames were automatically tracked using a conventional image processing algorithm.

### Procedure

Participants were instructed that they would alternately and repeatedly communicate with a female person and a female-type android robot. They were invited to the android room and had opportunities to see the actual appearance of the android prior to habituation. After allowing them to leave the room, an experimenter brought each participant into the android booth to calibrate the gaze detection device for him (note that during the calibration process, the android robot was concealed by a large white board placed in front of it). After calibration in the android booth, the same process was conducted in the human booth.

Participants then conducted five conversation trials in total alternately in each room. All sessions were conducted on the same day. Each trial always started from the human room to see if the looking pattern to a human interlocutor was enhanced through the repetition of the conversation with a human or an android. In each trial, either the android or the human started by greeting the participant and asking him to talk to it or her for a while. After repeating the question and answer conversation several times, the android or the human told the participant to move to the opposite booth or the outside of the room when all of the trials were over. We decided to start and end the experiment with human interlocutor sessions in order to evaluate whether the experiences of conversations with the android robot between these sessions resulted in a change of behavior toward human interlocutors. As a result, the number of the sessions with the human interlocutor was one more than the number of the sessions with the android.

### Dependent Variables and Statistical Analysis

We analyzed when the detected fixation points remained on the human’s or android’s face region in the captured images: when they looked at the interlocutor’s face. The AOI, the region defined by two connected half ellipses to cover the facial region of the interlocutors, was identified around their face by manual registration in the captured images. The radiuses of the ellipses were chosen to be 1.5 times larger than the face to successfully cover it against sensory noise. We calculated the looking-face ratio, the time ratio when the fixation points remained on the facial AOI with respect to the successful period of detection, for each session and calculated the average among sessions with the same interlocutors. The looking-eye bias was also calculated as the ratio of time when the subjects’ eye fixations stayed on the upper region of the AOI (i.e., approximately on the eyes) with respect to the time when they stayed within the AOI (face).

To analyze the differences in looking-face and looking-eye ratios, we considered subject type and interlocutor type factors. Thus, we adopted the analysis design of between subject (ASD or non-ASD group) and within subject (human or android interlocutor) ANOVA (note that when significant interaction was found, the simple main effect was tested using the pooled error term).

We also analyzed whether the looking-eye bias for the human interlocutor increased through the sessions. For each subject, we ran a simple least squares bivariate linear regression of the looking-eye ratio values for that subject on session as an ordinal variable. Then, the mean of these computed regression coefficients across persons in a given subject group was tested to ascertain if it was significantly different from zero by using one-sample *t*-test.

## Results

The 10 participants were able to engage in all five conversation sessions (see **Supplementary Table 1** for data analyzed in this paper). The total duration spent for the human and android sessions was 376.2 (SD = 70.1) [s] and 317.0 (SD = 39.3) [s] in the ASD group, respectively, and 363.8 (SD = 36.1) [s] and 325.1 (SD = 23.8) [s] in the non-ASD group, respectively. The average and standard deviation of the time length of each session for each group are shown in **Table 1**. The detection rates of fixation points, i.e., percentage of the periods when the gaze detector succeeded in capturing them, depended on whether the subject directed his gaze to the interlocutor because of the limitation of the measurable range of the unwearable type of gaze detection device. The average rate was 81.0% and 70.4% in the ASD and non-ASD group, respectively. In this study, we focused on the fixation patterns during these successful periods.

**Table 1:** Duration in seconds of each session for each group: The number is the mean value and that inside the brackets is its standard deviation.

Subject type	H1	A2	H3	A4	H5
ASD	140.2 (24.5)	169.9 (17.3)	122.4 (34.6)	147.1 (25.2)	113.6 (19.3)
Non-ASD	128.0 (11.2)	166.8 (9.3)	120.7 (16.9)	158.3 (15.3)	115.1 (14.1)

**Figure 3:**
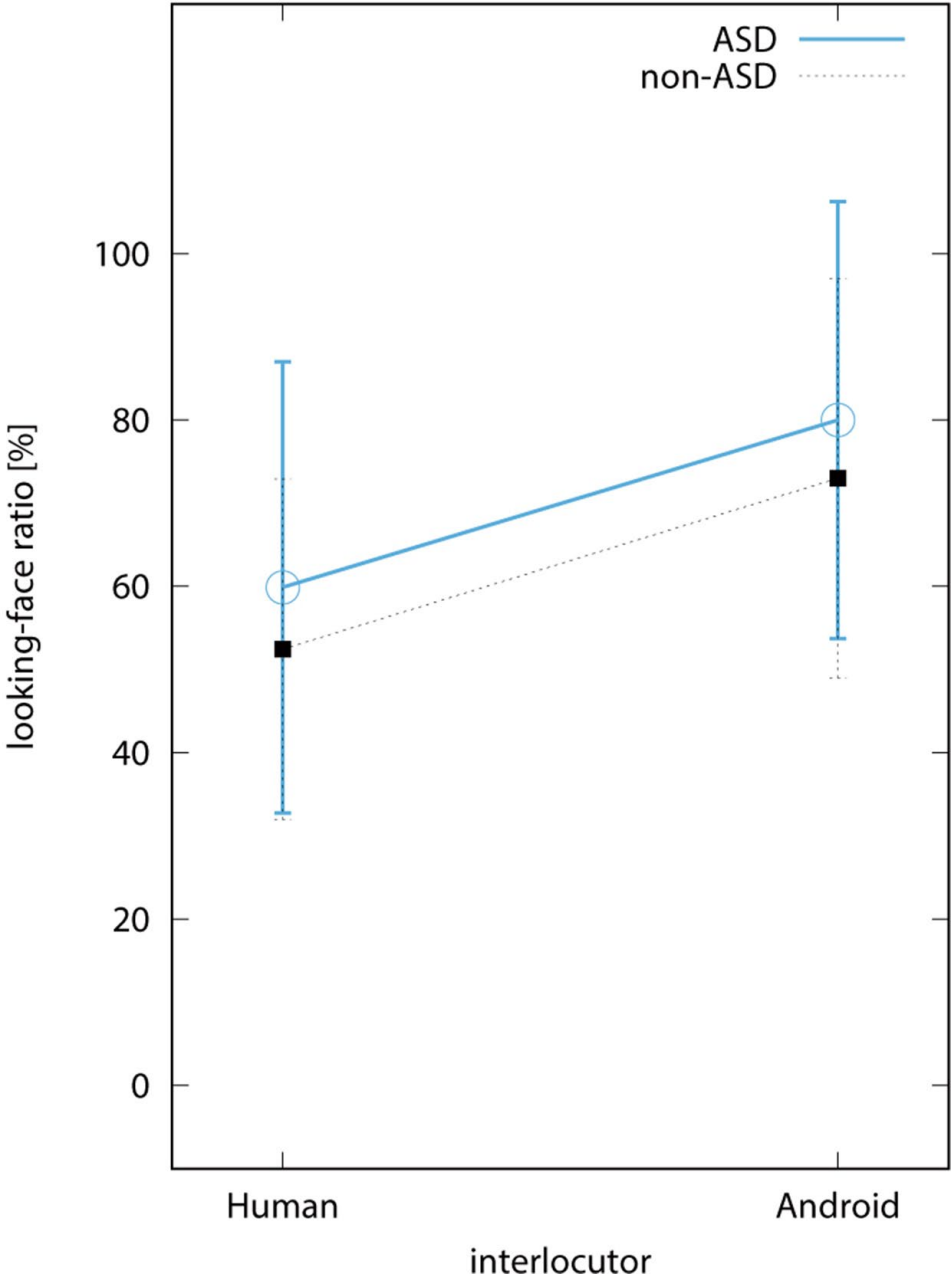
Looking-face ratio. The blue circular and black rectangular points indicate the average value among participants in the autism spectrum disorder (ASD) and non-ASD groups, respectively. The bars on the points are the standard deviations.

**Figure 3** shows the average looking-face ratio among sessions with the same interlocutors. In the ASD group, the average ratio in the human and the android conditions was 59.9% (SD = 27.7) and 80.0% (SD = 26.8), respectively. In the control group, it was 52.4% (SD = 25.6) and 73.0% (SD = 30.0), respectively. Two-way repeated measures ANOVA revealed the main effect of the interlocutor type [F(1,8) = 13.03, *p* < 0.01], while there was no significant interaction between factors of interlocutor type and the subject type [F(1,8) = 0.001, *n.s*]. This indicates that the subjects tended to look more at the face of the android robot than at that of the human interlocutor regardless of whether the subjects were with or without ASD.

**Figure 4:**
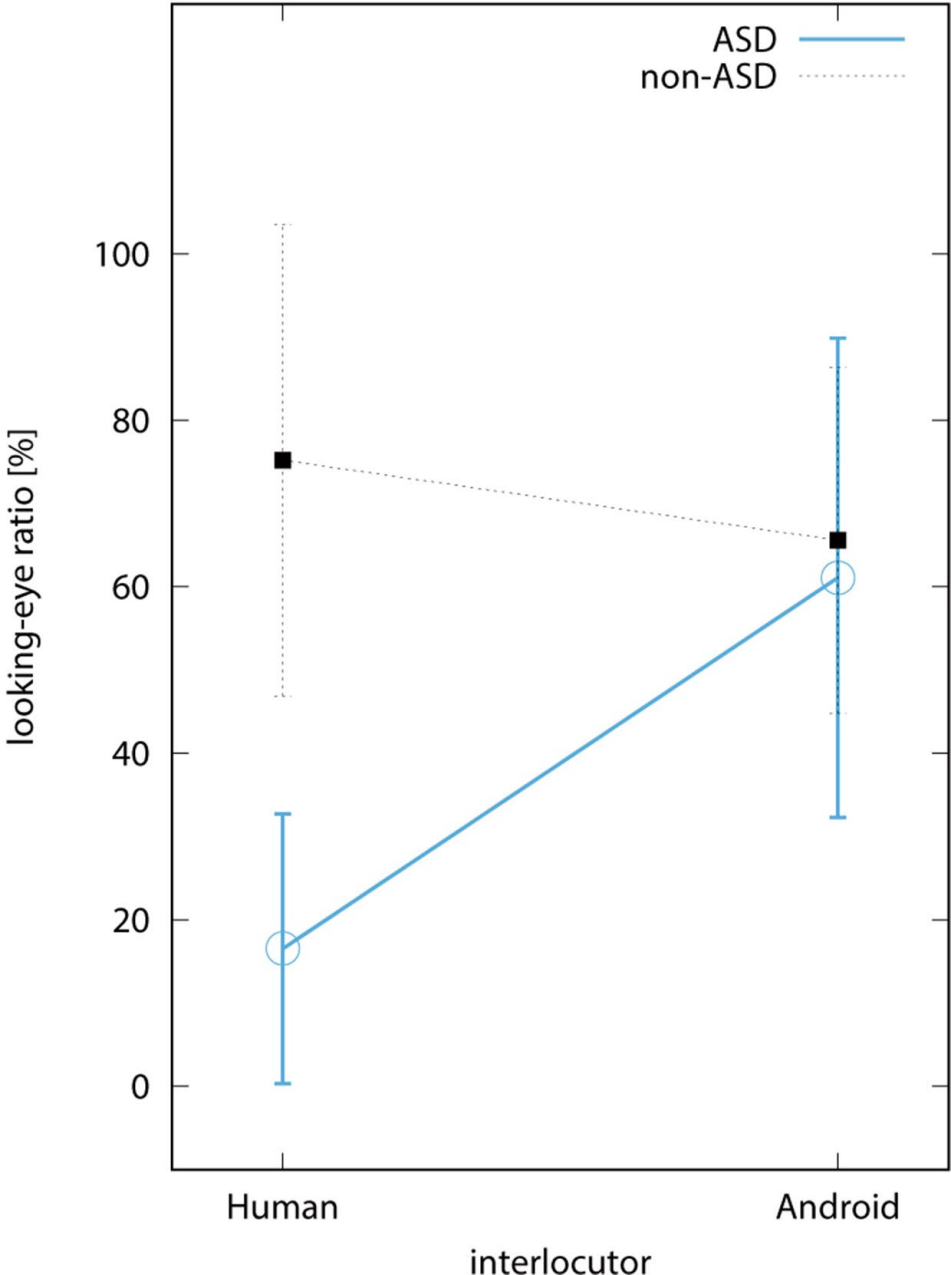
Looking-eye ratio. The blue circular and black rectangular points indicate the average value among participants in the ASD and non-ASD groups, respectively. The bars on the points are the standard deviations.

**Figure 4** shows the average looking-eye ratio among sessions with the same interlocutors. In the ASD group, the average ratio in the human and the android conditions was 16.5% (SD = 16.5) and 61.1% (SD = 29.4), respectively. In the control group, it was 75.2% (SD = 35.4) and 65.6% (SD = 26.0), respectively. Two-way repeated measures ANOVA revealed the significant interaction between factors of interlocutor and subject types [F(1,8) = 9.844, *p* < 0.05]. Subsequent analysis revealed a simple main effect of the interlocutor type in the ASD group [F(1, 16) = 10.13, *p* < 0.01] as well as a simple main effect of the subject type when the interlocutor was human [F(1,8) = 13.32, *p* < 0.01]. This indicates that individuals with ASD looked more at the area around the eyes of the android than that of the human and also looked less at that of the human than did the individuals without ASD.

**Figure 5:**
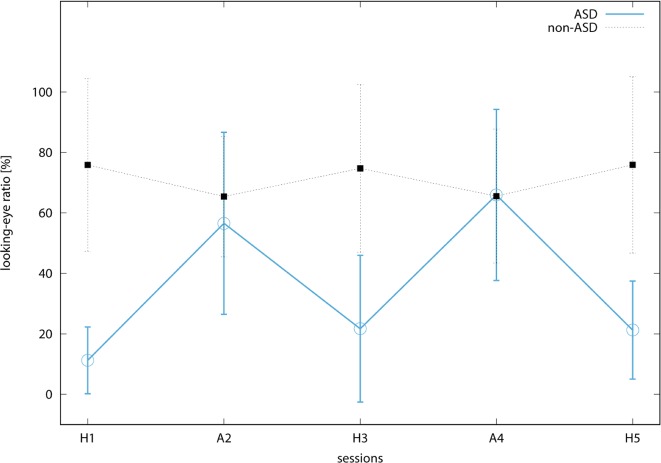
Transitions of looking-eye ratio along with sessions. The blue circular and black rectangular points indicate the average value among participants in the ASD and non-ASD groups, respectively. The bars on the points are the standard deviations.

**Figure 5** shows the transitions of the looking-eye bias along with the extension of the sessions. The salient M shape in the ASD group illustrates that the looking-eye bias is higher in the conversation with the android than with human. The average gradient of the looking-eye bias, which is calculated by fitting a coefficient of the bivariate linear model, in the ASD group was 0.039 (SD = 0.025) [%/session] while that in the non-ASD group was 8.3 × 10^−5^ (SD = 0.026). One-sample *t*-test revealed a marginally significant difference in that the gradient in the ASD group was more than zero (*t*(3) = 3.027, *p* < 0.1) while there was no significant difference in the non-ASD group (*t*(5) = 0.009, *n.s*).

## Discussion

In the current study, we conducted a single-day experiment to provide subjects with consecutive sessions in which they alternately talked to the human and android interlocutors five times in total and monitored how they looked at the eyes or faces of these interlocutors. Analysis of the detected fixation points allowed us to determine several features of participant looking patterns. Participants in both groups looked at the android more than at human interlocutors. ASD participants looked less at the human eye region than non-ASD participants and looked at the android eye region more than at the human eye region. Furthermore, although it is of only marginal significance, the time to look at the eye of the human interlocutor increased with increasing number of sessions in the ASD group.

Although it should be carefully interpreted due to the small sample size, the main effect of interlocutor type on the looking-face ratio suggests that persons look more at the face of an android than at that of another person in interlocutor-paced communication such as that given in this experiment, regardless of whether they had ASD. This may not be surprising as the curiosity for novel or wired objects (i.e., the android robot) likely led participants to do so. However, there is a possibility that this might also reflect the general easiness of the android robot for any or broader kinds of persons. Recent studies in the field of human–robot interaction report that adolescents with ASD and young adults with typical development might feel more at ease with a small desktop-type robot as an interlocutor when they are told to disclose their daily distress or autobiographical story (28). Further experiment after long-term habituation would be beneficial to consider this effect. Again, although it should be carefully interpreted due to the small sample size, the simple main effect of the interlocutor type on the looking-eye bias in the ASD group suggests that individuals with ASD do not show absence of eye contact in the face-to-face communication with the android robot, which is a typical diagnostic feature (2). However, the cause of the difference in participant looking patterns remains unclear, which presents a notable limitation of the current study.

Considering the fact that the voice quality of the android robot was recorded from and therefore identical with the paired human interlocutor, the perceptual difference of the two interlocutors is considered to stem from the visual modality. In this experiment, the facial expression of the android robot was minimally designed to reduce humanly features by making it produce only spontaneous eye-blinking and lip movement synchronized with the produced utterances. In other words, the facial movement of the android robot was designed to look calm or predictable by eliminating emotional expressions and gaze movement, which are usually dynamic during conversations (7, 29). It has been widely argued that individuals with ASD have limited or abnormal perceptual capability of social signals (30, 31). Kozima et al. argued that a robot that does not show human-like subtle expressions such as their small snowman-type robot has an advantage in keeping children with ASD interested in communication. On the other hand, human caregivers were considered to unconsciously produce too many subtle expressions that are difficult for the children to understand (10). Further experiments in which the modality and degree of the human-like expressions of the android robot are controlled is necessary to understand the extent to which human-like expressions should be reduced or can be added for providing individuals with ASD with opportunities to have social interaction. Such knowledge could be useful to improve the treatment and education supported by information technology such as e-learning for employment support using virtual humanoid agents (32, 33).

Analysis of the gradient of the looking-eye ratio along with sessions shows that it had a small yet significant increase in the ASD group. This increase was not observed in the non-ASD group. If we will obtain the significant result in the future experiment with more samples, it will be the first report indicating the possibility that individuals with ASD increase social behavior such as looking-eye in subsequent human–human conversation following human–robot conversation. Previous work using mechanical-looking humanoid robots instead of an android robot succeeded in promoting children with ASD to establish eye contact or learn skills of joint attention with the robot but did not report such a sign of generalization to human (11, 19). What could be the reasons for the weak but positive sign of such successful generalization of the promoted gaze-related behavior after the very short intervention in the current work? It is worth arguing for the contribution of two kinds of similarities between the android and human interlocutors. First, because the eyes of the android have quite similar visual properties to those of the human being, the increased tendency of attention to those of the android might be confused with those of the human without strong refusal at the perceptual level. Furthermore, such confusion in the sensorimotor system might be enhanced owing to the auditory likeness of the android and human interlocutors as the voice of the android was created by recording that of the human interlocutor. It should be confirmed whether or to what extent such similarities of android to human are necessary and can be scheduled for this potential change by further experiments using both android and a less human-like humanoid robot. In addition to such confusion in the sensorimotor system, the possibility of top-down modulation should be investigated. For example, participants in ASD group can become accustomed to the human interlocutor through the sessions, which, in turn, can inhibit the tendency of averting looking eyes. To examine this possibility, one should compare the results described herein with those obtained under the condition of experiencing only human sessions.

On the other hand, it is also worth noting that the current study focused on showing the possibility of improvement of a single measure (that is, looking-eye bias) by a single day intervention. In a series of studies by a pioneering group, multiweek interventions have already been conducted using a small humanoid robot and a mobile robot on some measures such as social attention (15), verbal communication (16), imitation and synchrony (17), and sensory behavior and affective states (18), with some negative results being obtained. Therefore, it should be important for future work to conduct longitudinal experimental interventions using the android robot on more than one measure and comparing these results with those from the previous studies using the humanoid robot.

Although the current study is limited by small sample size, we adopted a parametric method, ANOVA, for preliminary analysis to consider both between-subject (subject type) and within-subject (interlocutor type) variables, because, to the best of our knowledge, no appropriate established nonparametric test methods applicable to such mixed designs are available. Since the application of such a parametric test is prone to providing too robust results, they should be treated as preliminary ones. Furthermore, the experiment always started and ended with human sessions to explore whether the looking-eye ratio increased after the sessions with android robots. Therefore, it is necessary to conduct further experiments using larger samples while counterbalancing the order of sessions. Moreover, once future studies with larger samples confirm the increase of eye contact, it is worth examining whether this increase is further linked to the improvement in other social communicative deficiencies often seen in ASD patients such as turn-taking and conversational topic maintenance.

The control group of non-ASD individuals with a history of not adapting to school was used to remove the potential effects for those with this specific psychiatric element. The clinical team including an experienced child/adolescent psychiatrist recognized that individuals in the experiment group had ASD and had a history of not adapting to school. The same team recognized that individuals in the control group did not adapt to school (mainly because of bullying) but did not have ASD or other psychiatric disorders. In addition, the pediatric neurologist did not recognize any neurological disorders in all subjects. However, despite this careful assessment, it cannot be concluded that control-group participants did not have any other psychiatric symptoms such as attention difficulties at all. Therefore, we have to mention that the current study is limited by the risk that individuals in the control group had unrevealed psychiatric symptoms.

## Conclusion

The pilot experiment measuring the fixation pattern during the consecutive conversation with a human and an android robot suggested the possibility that the looking eyes of the interlocutor, which is one of the typical diagnostic feature of ASD, can be increased for the android robot. It is, however, necessary to note that these results should be limited by the small sample size. Future studies confirming to what extent the findings are maintained for many subjects in many types of robot experiments are necessary to understand whether or how we can expect robot technology to be used for the treatment and education of face-to-face communication. Furthermore, even if it is successful, mere promoting of the tendency to look at the eyes is not sufficient for treatment and education of social communication using eyes. The development of adequate contents to enable individuals with ASD to realize the importance or benefit of attending to eyes during the conversation as well as learn any further social skills or protocols that can be experienced only after attending to the eyes of other persons therefore needs to be considered.

## Ethics Statement

The current study was approved by the ethics committee of University of Fukui. Written informed consent was obtained from all participants included in the study and their guardians.

## Author Contributions

YY, HK, YM, MM, MK, and HI contributed to the conception and design of the study. YY, HK, and YM organized the experiment. YY performed the statistical analysis. YY wrote the first draft of the manuscript. YY and HK wrote sections of the manuscript. All authors contributed to manuscript revision and read and approved the submitted version.

## Funding

This work was supported in part by the JST ERATO ISHIGURO Symbiotic Human-Robot Interaction Project (JPMJER1401) and was partially supported by a Grants-in-Aid for Scientific Research from the Japan Society for the Promotion of Science (25220004, 15K12117) and The Center of Innovation Program from the Japan Science and Technology Agency, JST, Japan.

## Conflict of Interest Statement

The authors declare that the research was conducted in the absence of any commercial or financial relationships that could be construed as a potential conflict of interest.

## Supplementary Materials

Supplementary Video A sample video of the android robot talking to human. Note that parts of the voice were muted in the video, which might identify any individuals who attended to the experiment.

Supplementary Table 1 Data analyzed in this paper: age and CARS scores of the subjects, conversation time [second], time when the gaze is detected [second], ratio of the time when the gaze is detected, time when the participant is regarded to look at the region around face [second], ratio of participant’s looking at the interlocutor’s face, time when the participant is regarded to look at the region around eyes [second], ratio of participant’s looking at the interlocutor’s eyes, and the gradient of the ratio of participant’s looking at the interlocutor’s eyes along with sessions.
